# Study of PtO_x_/TiO_2_ Photocatalysts in the Photocatalytic Reforming of Glycerol: The Role of Co-Catalyst Formation

**DOI:** 10.3390/ma11101927

**Published:** 2018-10-10

**Authors:** Katalin Majrik, Zoltán Pászti, László Korecz, László Trif, Attila Domján, Giuseppe Bonura, Catia Cannilla, Francesco Frusteri, András Tompos, Emília Tálas

**Affiliations:** 1 Institute of Materials and Environmental Chemistry, Research Centre for Natural Sciences, Hungarian Academy of Sciences, Magyar tudósok körútja 2, H-1117 Budapest, Hungary; majrik.katalin@ttk.mta.hu (K.M.); paszti.zoltan@ttk.mta.hu (Z.P.); korecz.laszlo@ttk.mta.hu (L.K.); trif.laszlo@ttk.mta.hu (L.T.); tompos.andras@ttk.mta.hu (A.T.); 2 NMR Research Group, Research Centre for Natural Sciences, Hungarian Academy of Sciences, Magyar tudósok körútja 2, H-1117 Budapest, Hungary; domjan.attila@ttk.mta.hu; 3 National Council of Research—CNR-ITAE, “Nicola Giordano”, Via S. Lucia 5, 98126 Messina, Italy; giuseppe.bonura@itae.cnr.it (G.B.); catia.cannilla@itae.cnr.it (C.C.); francesco.frusteri@itae.cnr.it (F.F.)

**Keywords:** TiO_2_, co-catalyst, Pt(NH_3_)_4_(NO_3_)_2_, high temperature H_2_ treatment, high temperature N_2_ treatment, calcination, glycerol, H_2_ evolution

## Abstract

In this study, relationships between preparation conditions, structure, and activity of Pt-containing TiO_2_ photocatalysts in photoinduced reforming of glycerol for H_2_ production were explored. Commercial Aerolyst^®^ TiO_2_ (P25) and homemade TiO_2_ prepared by precipitation-aging method were used as semiconductors. Pt co-catalysts were prepared by incipient wetness impregnation from aqueous solution of Pt(NH_3_)_4_(NO_3_)_2_ and activated by calcination, high temperature hydrogen, or nitrogen treatments. The chemico-physical and structural properties were evaluated by XRD, ^1^H MAS NMR, ESR, XPS, TG-MS and TEM. The highest H_2_ evolution rate was observed over P25 based samples and the H_2_ treatment resulted in more active samples than the other co-catalyst formation methods. In all calcined samples, reduction of Pt occurred during the photocatalytic reaction. Platinum was more easily reducible in all of the P25 supported samples compared to those obtained from the more water-retentive homemade TiO_2_. This result was related to the negative effect of the adsorbed water content of the homemade TiO_2_ on Pt reduction and on particle growth during co-catalyst formation.

## 1. Introduction

Hydrogen is an important secondary energy source of the future [1,2,3] because it can be used in an environmentally friendly manner [4,5,6] and its chemical energy can be transformed to electricity very effectively by means of fuel cells. Photocatalytic hydrogen production is a promising approach for transforming solar energy into chemical energy for storage [7]. Recent efforts indicate that photo-induced reforming of alcohols on semiconducting oxides in the presence of water may be an efficient way of solar energy based hydrogen generation [8,9]. If the alcohol is obtained from biomass, a nearly closed carbon loop is possible as CO_2_ formed during the reforming reaction may be consumed for the source biomass growth. Since the photo-induced reforming is not restricted to simple alcohols, and considering that the increasing production of bio-diesel is accompanied with increasing production of glycerol by-product, the use of glycerol as feedstock of photocatalytical reforming reaction would be an exciting possibility [10] (1):

(1) $$C_{3}H_{8}O_{3}{+3H}_{2}O\overset{\mathrm{UV}-\mathrm{Vis}\;\mathrm{irradiation}}{\underset{\mathrm{Photocatalyst}}{\to }}{3\mathrm{CO}}_{2}{+7H}_{2}\;$$

Upon using aqueous solutions of glycerol at ambient conditions in the presence of Pt/TiO_2_ photocatalysts and a solar light-simulating source, it has been concluded that glycerol photoreforming may provide an efficient and low cost method for the production of renewable hydrogen [11]. While the conversion of the glycerol in the photocatalytic reforming reaction is of one or two-orders of magnitude less than that of other glycerol reforming systems at this moment, above reaction is widely studied and used as a model reaction to compare photocatalysts [12,13].

In any hydrogen-producing photocatalytic reaction, regardless of whether photocatalytic reforming of alcohols or overall water splitting is involved, significant activity can only be achieved if a proper co-catalyst is present on the semiconductor [14]. Consequently, the activity determining factors for these photocatalysts are the nature of the semiconductor, the co-catalyst, and the interaction between them.

Regarding the photocatalysts, TiO_2_-based materials are among the most frequently used ones because of their good stability and efficiency [15,16,17,18]. Different types of methods (sol-gel [19], precipitation [20], flame spray pyrolysis [21,22], etc.) are widely applied for preparation of TiO_2_. However, TiO_2_ samples from different sources show very different photocatalytic activities [23] as specific surface area [24], morphology [25], size of nanoparticles [26,27], rutile/anatase/brookite ratio [24,28], vacancy structure [29], type and amount of surface OH groups [30], etc. are different and are all able to influence the photocatalytic behavior.

Regarding the co-catalyst, the activity of TiO_2_ in a photoinduced reforming reaction can be increased with at least an order of magnitude in its presence [12,31,32,33]. The advantages of the co-catalysts can be attributed to the reduced charge recombination as a result of promoted charge separation and transport driven by junctions/interfaces [12,34]. Another important role of the co-catalyst is to provide reaction sites for elementary reaction steps subsequent to light absorption, such as the formation of molecular hydrogen and its desorption from the surface [12,34]. Metal nanoparticles, especially those containing Pt, are very effective co-catalysts for H_2_ production [12,31,35].

In order to load metal nanoparticles on the surface of the semiconductors, several different methods are available. Commonly used techniques include in situ photodeposition [36,37] and deposition of pre-prepared metal colloids [38,39]. A traditional way for preparing supported metal nanoparticles is impregnation with the appropriate metal precursor followed by high temperature heat treatment in hydrogen [40]. Calcination of the metal precursor loaded on semiconductors by incipient wetness impregnation has also resulted in effective co-catalysts for H_2_ production in the methanol photocatalytic reforming reaction [29,41]. In case of co-catalyst formation by calcination, in situ reduction of platinum has been found during the photoinduced H_2_ production from methanol-water reaction mixture [29,42]. Processes such as the effect of the irradiation, reduction by the reactant (methanol) and/or by the reaction product (in situ formed hydrogen) were considered as possible reasons of the reduction of the calcined photocatalysts under the reaction conditions [29].

The most frequently applied commercially available platinum precursors are chloroplatinic acid hexahydrate (H_2_PtCl_6_·6H_2_O) [43,44,45], tetraammineplatinum(II) chloride hydrate (Pt(NH_3_)_4_Cl_2_·H_2_O) [46,47], platinum acetylacetonate (Pt(C_5_H_7_O_2_)_2_) [44,48], and tetraammineplatinum(II) nitrate (Pt(NH_3_)_4_(NO_3_)_2_ [49,50,51,52]. Nowadays, the latter is increasingly used because of its high solubility in water and because it allows the formation of small platinum nanoparticles on the support; furthermore, all ligands can be removed from it upon heating the Pt(NH_3_)_4_(NO_3_)_2_ impregnated sample. Accordingly, Pt(NH_3_)_4_(NO_3_)_2_ is the metal precursor used for synthesis of heterogeneous catalytic systems active in reforming n-hexane [53], ethylene glycol [54], methane [55], hydrodeoxygenation of 5-hydroxymethylfurfural [56], deoxygenation of fatty acids and their esters [49], reverse water gas shift reaction [57], Fischer-Tropsch synthesis [51], hydrogenolysis of glycerol [52], etc.

However, in order to obtain supported metal particles with suitable size for a catalytic reaction, an appropriate decomposition procedure of the metal precursor is necessary. Specifically, thermal decomposition of Pt(NH_3_)_4_(NO_3_)_2_ was studied from the early times [58] and decomposition of the [Pt(NH_3_)_4_]^2^^+^ ion introduced into zeolites by ion exchange or impregnation was also studied in detail [59,60,61,62,63,64,65,66]. In case of zeolites, water has been found to play a decisive role in the decomposition of the metal precursor. Based on UV spectroscopic studies, possible formation of [Pt(NH_3_)(H_2_O)_x_]^2+^ has been suggested in the first step of oxidative decomposition of [Pt(NH_3_)_4_]^2^^+^ in HZSM-5 [62]. Based on temperature programmed reduction (TPR) and re-oxidation results, it has been concluded that Pt^2+^ ions after losing their NH_3_ ligands react with H_2_O leading to PtO microcrystallites in NaY [60]. It has also been found that small Pt particle size and narrow particle size distribution is obtained in zeolites only by the use of very slow (e.g., 0.1–0.2 °C/min) heating rates during the calcination before the final hydrogen treatment. When [Pt(NH_3_)_4_]^2^^+^ was loaded on a mesoporous support, the Pt dispersion was less sensitive to the calcination heating rate [66] due to the higher desorption rate of ammonia and water from it compared to that from the zeolite microporous structure. Literature studies report that, upon using inert atmosphere during the decomposition of [Pt(NH_3_)_4_]^2^^+^ in zeolites, auto-reduction of platinum occurs [59,62] and the Brønsted acid sites can react with the liberated ammonia [62,63].

In case of the Al_2_O_3_ support, it has been found that ammonia ligands adsorb on the acid sites of the surface after breaking of the Pt^2+^-N coordinative bonds and they remain there until the temperature is high enough to allow desorption from the strongly acid sites [67]. Based on mass spectrometry data, it has been suggested that ammine complex disruption, ammonia evolution, and Pt^2+^ reduction are three separate processes that do not take place simultaneously. It has been also found that the direct reduction of [Pt(NH_3_)_4_]^2^^+^ on alumina at 350 °C yields the biggest metal particles (35 Å), while calcination before reduction produces a much higher dispersion (metal particle diameter 10 Å) [67].

When Pt/silica catalysts are prepared by adsorption of [Pt(NH_3_)_4_]^2+^ from strongly basic impregnation solutions, hydroxyl groups of silica are deprotonated, and platinum containing cations are readily deposited on the support, via strong electrostatic adsorption (SEA) [68]. It has been found that the pH not only determines the amount of adsorbed Pt, but also influences the particle size of the reduced catalyst. The highest dispersion is obtained at lower Pt loading and drying in air at 100 °C, followed by reduction in H_2_ at 250 °C [68]. While the SEA approach seems to be more effective method for the preparation of highly dispersed silica supported platinum than the incipient wetness method, the latter is also successful at low metal loads (0.5–1%) [68]. By means of combination of HRTEM, mass spectrometry, and Quick EXAFS, Oudenhuijzen and coworkers have explored the reactions taking place under Pt(NH_3_)_4_(NO_3_)_2_ decomposition in different gas flows such as H_2_, O_2_ and Ar/He [69]. They have reported that autoreduction of silica supported Pt(NH_3_)_4_(NO_3_)_2_ in inert atmosphere results in metal-particle size smaller than that obtained by hydrogen reduction or by hydrogen reduction after calcination [69]. They described that the key to making the smallest particles with the narrowest particle-size distribution is to avoid the formation of mobile species able to precipitate as metal clusters [69]. 

During the preparation of high-surface-area carbon black supported platinum electrocatalysts with 60% Pt content, the use of Pt(NH_3_)_2_(NO_3_)_2_ resulted in larger particle size than H_2_PtCl_6_ [70]. At the same time, a strong exothermic effect has been observed during the decomposition of Pt(NH_3_)_2_(NO_3_)_2_ [70], which probably was not obvious at low metal load [69].

The aim of this work was to explore the main relationships between the structural properties of PtO_x_/TiO_2_ photocatalysts and their photocatalytic behavior in the glycerol photocatalytic reforming reaction. Several different PtO_x_/TiO_2_ photocatalysts were prepared using Pt(NH_3_)_2_(NO_3_)_2_ precursor by exploiting different co-catalyst formation methods such as reduction by high temperature hydrogen treatment, autoreduction in inert atmosphere (nitrogen), and calcination in air. Two different types of TiO_2_ were used as support.

In analyzing the results, the attention was focused on the platinum chemical state in the co-catalyst as well as on the co-catalyst-TiO_2_ interaction. In order to obtain more information about the real working catalyst, chemico-physical characterization results of fresh and recovered samples were compared.

## 2. Materials and Methods

### 2.1. Materials

Titanium-isopropoxide (≥97.0%, Sigma-Aldrich Inc., St. Louis, MO, USA) was used for the synthesis of homemade TiO_2_. Aerolyst^®^ TiO_2_ (P25, Evonik, Essen, Germany) was also used as semiconductor. The Pt(NH_3_)_4_(NO_3_)_2_ platinum precursor was supplied by Sigma-Aldrich Inc. (St. Louis, MO, USA). Glycerol (99%), 2-propanol and HNO_3_ (65%) were products of Molar Chemicals Ltd. (Budapest, Hungary. Hydrofluoric acid (38%) and boric acid were products of Reanal (Budapest, Hungary). Absolute ethanol was purchased from VWR International (Fontenay-sous-Bois, France). Double distilled water (18 MΩ) was used for the synthesis of photocatalysts and for preparation of glycerol solution. The gases (H_2_, N_2_, Ar) used in this work were products of Linde Gáz Magyarország Zrt. (Budapest, Hungary) with 5.0 purity. Special mixture of 5% H_2_ in N_2_ for calibration of GC was bought from Messer Hungarogáz Ltd. (Budapest, Hungary).

### 2.2. Synthesis of Photocatalysts

The homemade TiO_2_ was prepared by the precipitation-aging method during which typically 3.5 cm^3^ titanium-isopropoxide was added dropwise to 95 cm^3^ aqueous solution of HNO_3_ (3.5%) with continuous agitation to obtain transparent TiO_2_ sol. The synthesis mixture was stirred for 4 days, then it was heated up to 65 °C in order to evaporate the solvent under continuous stirring. Finally, the powder was dried in an oven overnight at 80 °C followed by calcination at 400 °C. The abbreviated name of this TiO_2_ was PA. The P25 TiO_2_ was used for impregnation without further treatment.

**Figure 1:**
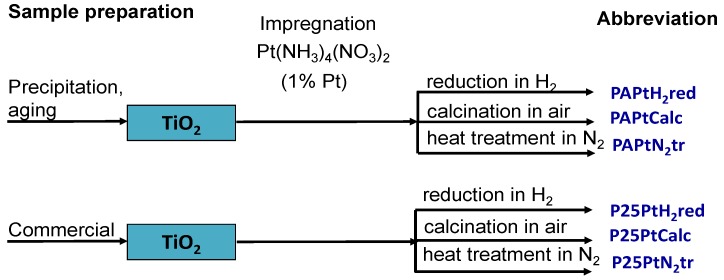
Denomination of the various PtO_x_/TiO_2_ catalysts.

Platinum was introduced from aqueous solution of Pt(NH_3_)_4_(NO_3_)_2_ by incipient wetness impregnation. The nominal platinum load was 1 wt %. The treatments of dried samples were: (i) Calcination for 1 h at 300 °C in air after heating up to 300 °C with 1 °C/min heating rate (PtCalc samples); (ii) reduction for 1 h at 400 °C in H_2_ flow by using a heating rate of 5 °C/min after heating up in N_2_ to 150 °C with β = 5 °C/min (PtH_2_red samples); and (iii) treatment for 1 h at 400 °C in N_2_ flow with β = 5 °C/min (PtN_2_tr samples). The lineage of the various PtO_x_/TiO_2_ catalysts is depicted in Figure 1.

The real Pt content of the photocatalysts measured by ICP-OES technique after microwave assisted dissolution in 1:8 mixture of concentrated nitric acid (65%) and hydrofluoric acid (38%) were 0.97 and 0.80 wt % Pt for PAPt and P25Pt samples, respectively. The Pt content of the recovered samples did not decrease.

### 2.3. Photocatalytic Hydrogen Generation

The photocatalytic reaction was carried out in a reactor system of 10 quartz glass units equipped with magnetic stirrers, gas inputs and outputs as described before [29]. The size of the cylindrical quartz glass units were 140 mm in height and 60 mm in diameter. Nitrogen gas with 20 cm^3^/min flow rate was continuously bubbled through all reactor units in parallel. According to blank experiments, all reactor units were equivalent in terms of the catalytic activity. In case of kinetic measurements, one channel mode was used. The initial concentration of glycerol was 6 *v*/*v*% in distilled water. The reaction was carried out at room temperature. The amount of catalyst and the reaction volume in every unit was 0.100 g and 260 cm^3^, respectively. Osram HQL de luxe 125 W lamps were used as light sources operated in the UV-visible region. A GC (Agilent 7820A, Agilent Technologies, Santa Clara, CA, USA) equipped with SUPELCO Carboxen 1010 column (Supelco Analytical, Bellefonte, PA, USA) and TCD detector was used to follow the H_2_ production. The internal standard of the GC analysis was argon gas added to the vapor-gas mixture before the GC sampling valve. The H_2_ production was expressed as H_2_ production rate (mmol/h). The reaction was monitored for 270 min. After the photocatalytic reaction, the samples were recovered from the aqueous glycerol solution by centrifuging and washed with 3 × 50 cm^3^ absolute ethanol followed by drying under N_2_ flow.

### 2.4. Chemico-Physical Characterization of Photocatalysts

Nitrogen physisorption measurements were carried out at −196 °C using Thermo Scientific Surfer automatic volumetric adsorption analyzer (Thermo Fischer Scientific, Berlin, Germany). The specific surface area was calculated by the BET method in the range of relative pressures from 0.05 to 0.30. The pore-size distributions were calculated from desorption isotherms by the BJH method. Before the analysis, TiO_2_ samples were outgassed under vacuum for 2 h at 250 °C.

Temperature programmed desorption (NH_3_-TPD) experiments were performed using Autochem 2920 (Micromeritics, Norcross, GA, USA) equipment with QMS (Thermostar, Pfeiffer Vacuum, Asslar, Germany) analysis. The samples were pretreated at 150 °C in helium flow for 1 h, followed by NH_3_ adsorption to saturation and helium purge at 50 °C for 1 h. Desorption was performed by a temperature ramp in helium between 50 and 600 °C at the heating rate of 5 °C/min.

Diffuse reflectance UV-visible spectra of the samples were registered using a Jasco V-570 UV-Vis (Jasco, Tokyo, Japan) spectrophotometer equipped with a NV-470 type integrating sphere. The data were collected between 300 and 800 nm wavelength intervals with 100 nm/min speed.

X-ray powder diffraction (XRD) patterns were obtained in a Philips model PW 3710 based PW 1050 Bragg-Brentano parafocusing goniometer (Philips, Eindhoven, The Netherlands) using CuK_α_ radiation (λ = 0.15418 nm), graphite monochromator, and proportional counter. Silicon powder (NIST SRM 640) was used as an internal standard and the scans were evaluated with profile fitting methods. Reference cards from the ICDD PDF-2 (1998) database were used. Crystallite sizes were calculated from reflection line broadening using the Scherrer-equation.

A Philips CM12 (Philips, Eindhoven, The Netherlands) instrument equipped with a high-resolution camera was used to acquire and elaborate the TEM images. Powdered samples were dispersed in 2-propanol under ultrasound irradiation and the resulting suspension; dropwise was deposited on a holey carbon-coated support grid.

The ESR experiments were performed with a Bruker Elexsys E500 X-band spectrometer (Bruker, Rheinstetten, Germany). A typical microwave power of 1 mW and 1 G magnetic field modulation at ambient temperature were used. The magnetic field was calibrated with an NMR field meter. Signal intensity, linewidth, and g-factor (spectroscopic splitting factor) values were used to characterize the samples. The knowledge of the g-factor can give information about a paramagnetic center’s electronic structure.

The simultaneous thermogravimetric and mass spectrometric evolved gas analyses (TG-MS) were recorded on a Setaram LabsysEvo (Setaram, Lyon, France) thermal analyzer, in high purity (99.9999%) helium atmosphere, with a flow rate of 80 cm^3^/min. The measurements were done with a heating rate of 20 °C/min, in the temperature range of 20–600 °C. The obtained results were baseline-corrected, and then evaluated with the thermal analyzer’s processing software (AKTS Calisto Processing, ver. 1.43). Parallel with the thermogravimetric measurements, the analysis of the evolved water was carried out on a Pfeiffer Vacuum OmniStar™ (Pfeiffer Vacuum, Asslar, Germany) gas analysis system coupled to the above-described TGA. The gas splitter and transfer line to the mass spectrometer was preheated to 260 °C. Three masses were scanned (the molecular ion of water, *m*/*z*—18, the OH ion, *m*/*z*—17 and the atomic oxygen ion, *m*/*z*—16), with a scan speed of 100 ms/mass. The mass spectrometer was operated in an electron impact mode.

X-ray photoelectron spectroscopy (XPS) measurements were carried out using an EA125 electron spectrometer manufactured by OMICRON Nanotechnology GmbH (Taunusstein, Germany). The photoelectrons were excited by non-monochromic MgK_α_ (1253.6 eV) radiation. Spectra were recorded in the Constant Analyzer Energy mode of the energy analyzer with 30 eV pass energy resulting in a spectral resolution around 1 eV. For XPS experiments, the samples in the form of fine powder were suspended in isopropanol. Drops of this suspension were placed on standard OMICRON sample plates; after evaporation of the solvent, catalyst coatings with sufficient adhesion and electric conductivity were obtained. Effects of possible electric charging were compensated by adjusting the binding energy of the Ti 2p_3/2_ peak to 458.8 eV (literature value for TiO_2_). By this choice the O 1s binding energies coincided with the range expected for TiO_2_, and the leading component of the C 1s spectra arising from hydrocarbon contamination appeared around 284.6–284.8 eV, confirming the reliability of the calibration. Chemical states of the elements were deduced from high-resolution spectra using XPS databases [71,72]. Quantification was performed using combination of CasaXPS [73] and XPSMultiQuant [74,75].

## 3. Results

### 3.1. Photocatalytic Hydrogen Production from Glycerol over PtO_x_/TiO_2_ Catalyst Systems

**Figure 2:**
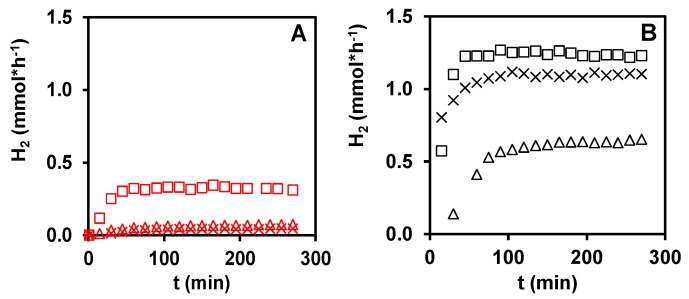
H_2_ production from glycerol over PtO_x_/TiO_2_ catalyst systems: (**A**) PA; (**B**) P25. □—co-catalyst formation by high temperature H_2_ treatment (PtH_2_red); ×—co-catalyst formation in N_2_ treatment under conditions of autoreduction (PtN_2_tr); and Δ—co-catalyst formation by calcination (PtCalc).

The results of the photoinduced reforming reaction of glycerol over different types of PtO_x_/TiO_2_ photocatalysts are shown in Figure 2.

In the absence of irradiation, no H_2_ production was observed. The H_2_ formation rate trend is characterized by an initial progressive increase followed by a plateau. The gradual increase in the hydrogen production rate in the initial period can be explained by considering two factors: (i) the achievement of glycerol liquid phase/nitrogen equilibrium; and (ii) the in situ progressive formation of catalytically active sites. For example, the in situ appearance of Pt^0^ observed in case of the co-catalyst formation by calcination was favorable for the hydrogen production in the photocatalytic reaction of methanol [29,42]. Regarding the recyclability of these catalysts, it can be said that the activity of the recovered P25PtH_2_ red sample did not decrease while that of P25PtCalc decreased with ~15% in the second cycle. However, in case of fresh P25PtCalc, a very slow increase of reaction rate can be observed even after four h reaction time. When we doubled the reaction time from 270 min to 540 min using fresh P25PtCalc in a new experiment the reaction rate further increased (from 0.64 mmol/h to 0.69 mmol/h).

From the results shown in Figure 2, it can be seen that the activity of photocatalysts based on P25 TiO_2_ is much higher than that of the PA based ones, regardless to the type of the co-catalyst formation treatment. One of the tasks of this work is to explain this behavior. The obvious answer is in the different structural quality of the P25 based TiO_2_ compared to that of the homemade one. However, the ratios of the H_2_ evolution rates of the P25-PA pairs were 2.5; 5.5; 14.9 for PtH_2_red, PtCalc, PtN_2_tr samples respectively, at 270 min. This result suggests that other physico-chemical properties than the structural features of the parent semiconductor should also be considered to justify such behavior. For example, the characteristics of the platinum co-catalysts in terms of oxidation state and particle size, which can change during the co-catalyst formation and during the photocatalytic reaction, could play a relevant role. In addition, by considering previous results, which showed in situ reduction of platinum during the photocatalytic reforming of methanol on the samples formed by calcination [29], it is important to understand if the in situ reduction of platinum occurs during the photocatalytic reforming of glycerol and to what extent.

In an attempt to answer these questions, a comparison of the characterization results obtained by XPS and TEM techniques of fresh and recovered sample pairs was carried out and the results are reported below. Moreover, since the literature data indicate a key role of the water for the decomposition of Pt(NH_3_)_4_(NO_3_)_2_ in the zeolites-based systems, the effect of residual water during the co-catalyst formation in the case of the different TiO_2_ semiconductors was evaluated and discussed too.

### 3.2. Characterization of Bare TiO_2_

In order to explore the effects of the structure of the semiconductor, the co-catalyst and the interplay between them on the hydrogen formation rate, first the data obtained by structure-sensitive techniques on the bare TiO_2_ supports are presented. Main structural properties, in terms of specific surface area (SSA), pore volume, maximum pore diameter, average particle size, and crystalline phases of the studied TiO_2_ are summarized in Table 1. 

One of the most striking differences between the two TiO_2_ materials appeared in the SSA values being much higher for the homemade sample, while pore volume and maximum pore diameter of that was significantly smaller. Another important difference between the two TiO_2_ samples was the phase composition: while P25 mainly contains anatase, PA mainly consists of the rutile phase along with brookite in comparable amounts to anatase. In accordance with the literature [15], the crystallite size of anatase or brookite is smaller than that of rutile. 

**Figure 3:**
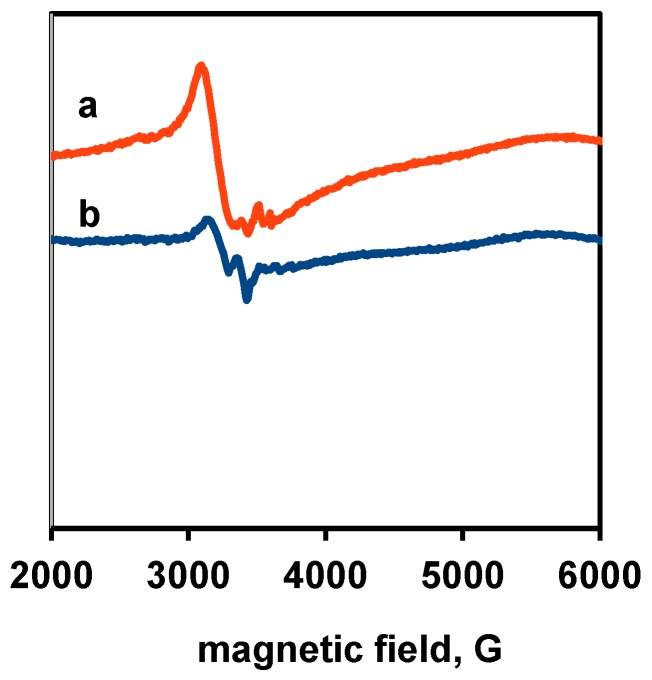
ESR spectra of the bare TiO_2_ samples: line (**a**) PA; and line (**b**) P25.

The ESR spectra of the two bare TiO_2_ samples, shown in Figure 3, reveal that the samples are characterized by similar defect structure. However, the very intense, sharp signal at about 3500 G (g = 2.003) observed in the ESR spectra of TiO_2_ prepared by sol-gel method [29] and attributed to electrons trapped in oxygen vacancy was absent in the semiconductors studied in this work. 

Anyhow, as already described in our previous work, these latter vacancies were unfavorable for photocatalytic reaction [29].

**Figure 4:**
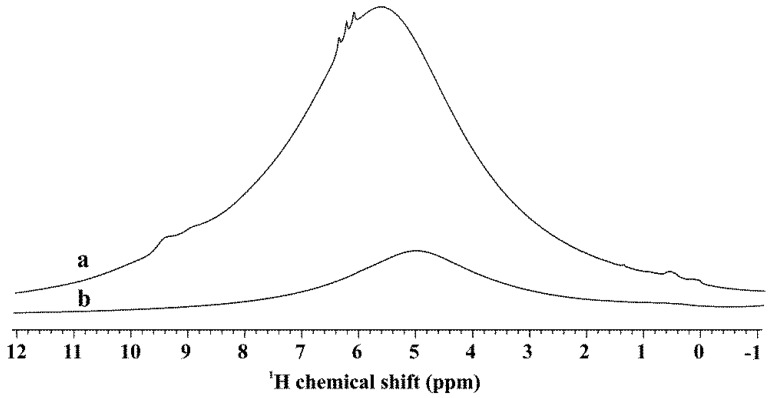
^1^H MAS NMR spectra of the bare TiO_2_ samples: line (**a**) PA; and (**b**) P25 (a: 0.055 g; b: 0.056 g); external reference: PDMS (0.085 ppm).

The ^1^H MAS NMR spectra of PA is characterized by an intense band that overlaps with other different peaks (Figure 4), resulting in a more complex profile than that of P25. While the main contribution was given by bridged –OH and H_2_O [29] in both samples, the intensity of that signal was much higher in PA than in P25.

Taking into account that the SSA value of PA is 2.3 times larger than that of P25, detection of larger amounts of adsorbed water was obvious [76], but such a great difference was not expected. This observation suggested that air exposed PA contains more H_2_O (and bridged –OH) per surface unit than P25. Furthermore, this signal appeared at somewhat higher chemical shifts in PA than in P25 suggesting stronger H-bonded structure of H_2_O molecules. Besides the main peak at 5.69 ppm, small intensity sharp (6.13, 6.27, 6.4 ppm) and broader (8.98 and 9.45 ppm) peaks can be observed. These additional signals indicate strong H-bonded sites in this sample. These results suggest a more acidic character for PA in comparison to P25. For the sake of completeness, it should be mentioned that certain amounts of H at low chemical shifts of 0.13 and 0.60 ppm, i.e., in the region of relative basic type of terminal OH groups also appeared in both samples.

According to the results of NH_3_-TPD measurements, the amount of desorbed ammonia was four times larger from PA than from P25. The SSA normalized values still showed noticeable differences. These observations are in accordance with the NMR results. 

**Figure 5:**
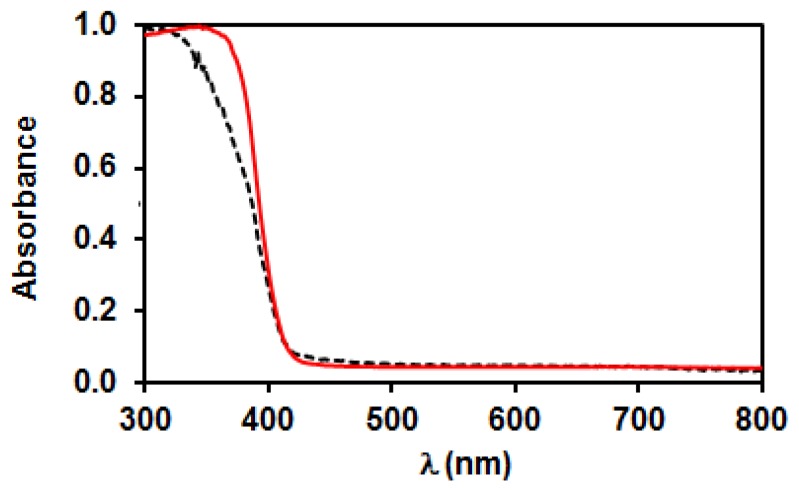
Diffuse reflectance UV-Vis spectra of the bare TiO_2_ samples: Solid line—PA; dashed line—P25.

Diffuse reflectance UV-Vis spectra of the two TiO_2_ samples also differ somewhat; the adsorption edges were at about 400 nm (Figure 5), but the homemade TiO_2_ showed somewhat higher absorbance in the near UV range. The Tauc plots suggested an indirect band gap with 3.00 eV width for P25, while a direct gap of 3.17 eV was found for PA.

The described properties of P25 are in line with those reported in literature [77]. It is known that P25 is a TiO_2_ material with high purity (99.5%) and narrow pore size distribution; its favorable properties are derived from its high temperature manufacturing process [78].

The above results obtained from different characterization methods clearly indicate that the increased specific surface area of the PA homemade TiO_2_ is accompanied by a more complex structure and more pronounced water retention ability compared to P25. The consistently lower photocatalytc activities found in glycerol reforming indicate that this complexity negatively influences the photocatalytic properties in both direct and indirect ways. Analyzing the activity on both bare TiO_2_ supports would help to interpret the differences in the behavior of the PtO_x_/TiO_2_ samples. However, in the absence of a metal co-catalyst, the H_2_ production of the bare supports is well below the detection limit in our system.

### 3.3. Physico-Chemical and Structural Characterization of Platinum Co-Catalysts

It should be mentioned that the introduction of Pt, followed by high temperature co-catalyst formation, only slightly changed the crystalline phase composition of the support. The evaluation of XRD patterns (not shown) of the homemade PA based PtO_x_/TiO_2_ samples revealed that the ratio of the rutile phase increased somewhat after the co-catalyst formation, while the ratio of brookite and anatase decreased slightly. For example, in the PAPtCalc sample, the percentages of rutile, brookite, and anatase phases changed from 54% to 63.6%, from 23% to 16.4% and from 23% to 20.0%, respectively. The extent of the co-catalyst formation induced phase transformation was very similar in the other two cases. Such results are in accordance with literature evidences obtained over P25 based samples [79], where a slight increase of the contribution from the rutile phase in both oxidation-treated and reduced P25 based Pt/TiO_2_ was observed in comparison to that of the parent P25. That change was ascribed to the dehydroxylation effect favored by the presence of Pt, which promotes rutile phase formation [79].

The platinum co-catalysts have been investigated in details by X-ray photoelectron spectroscopy. In order to get insight into the processes occurring during the photocatalytic reaction, data were collected on both the fresh catalysts and on those recovered after the photocatalytic experiment. The focus was on comparing the amount and the state of platinum in fresh and recovered samples. The Pt 4f profiles were first corrected for the charge-transfer satellite of the Ti 3s peak (which exactly overlaps with the Pt 4f region and appears shifted towards higher binding energies by 13.3 eV from the parent Ti 3s peak). Then, the spectra were fitted with a combination of two or three contributions: (i) spin-orbit doublet of metallic Pt with an asymmetric line shape derived from that of a reduced 40 wt % Pt/C catalyst with leading component (Pt 4f_7/2_) around or below 71 eV; (ii) spin-orbit doublet of Pt^2+^ (PtO or Pt(OH)_2_ with the Pt 4f_7/2_ component around 72–72.5 eV and (iii) spin-orbit doublet of Pt^4+^ (PtO_2_) with the Pt 4f_7/2_ component at 74.5–75 eV, if needed [72,80].

It should be mentioned that, exclusively, the presence of Ti^4+^ was observed in all investigated samples.

**Figure 6:**
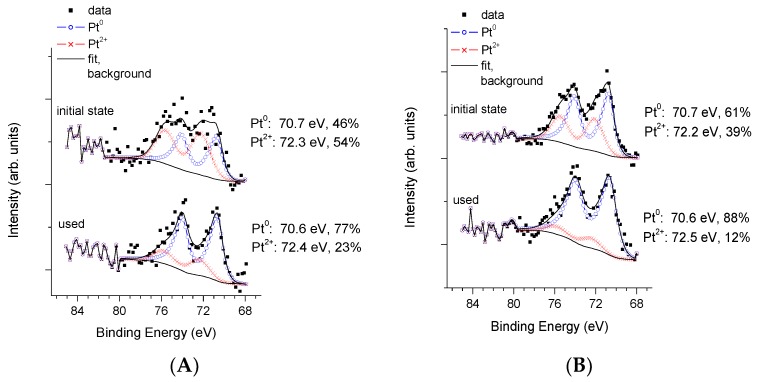
XPS study of fresh and recovered sample pairs in case of co-catalyst formation by H_2_ treatment at 400 °C: (**A**) PA; and (**B**) P25.

In Figure 6, Pt 4f spectra of the PAPt and the P25Pt fresh and used samples formed by high temperature hydrogen treatment are shown.

In both samples, the presence of Pt^0^ and Pt^2+^ is detectable. The binding energy of the metallic Pt 4f_7/2_ component is unusually low (around 70.6 eV), which can be attributed to charge transfer from the TiO_2_ towards the metal particle. Even if one expects that the high temperature hydrogen treatment reduces Pt to the metallic state, the presence of Pt^2+^ is not surprising taking into account the air exposure of sample during storage and transferring to the XPS chamber. According to XPS results, spectra of fresh and recovered P25PtH_2_red as well as fresh and recovered PAPtH_2_red did not significantly differ from each other, although a more reduced state after the photocatalytic experiment is evident in both cases and the Pt seems to be somewhat more oxidized in the PA TiO_2_ supported sample (Pt^2+^, 54% in fresh and 23% in used samples respectively) than in its P25 TiO_2_ supported counterpart (Pt^2+^, 39% in fresh and 12% in used samples, respectively).

The Pt/Ti ratio in the fresh samples was smaller in PAPtH_2_red (0.004) than in P25PtH_2_red (0.010). Then, it slightly decreased in the recovered in P25PtH_2_red (0.008), while it did not change in the recovered PAPtH_2_ red, as compared to the fresh ones. In terms of % weight, the Pt content of the PA supported sample was around 1.1%, while in the P25 supported sample, it was approximately 2.0% in the initial state.

**Figure 7:**
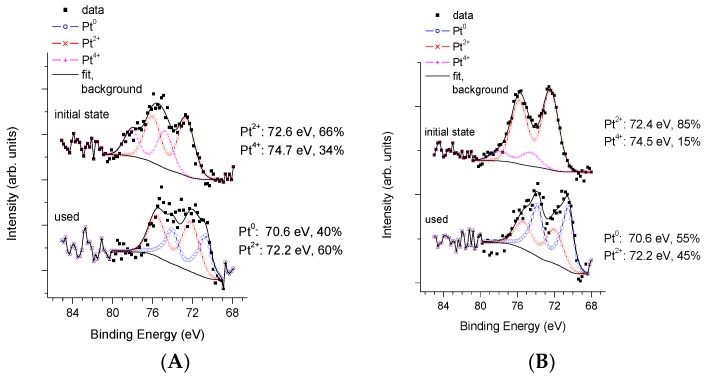
XPS study of fresh and recovered sample pairs in case of co-catalyst formation by calcination: (**A**) PA; and (**B**) P25.

In case of co-catalyst formation by calcination, the fresh PAPtCalc (pattern “initial state” in Figure 7A) sample contained only Pt^2+^ (66%) and Pt^4+^ (34%), while in the fresh P25PtCalc sample (pattern “initial state” in Figure 7B) more Pt^2+^ species (85%) and a smaller amount of Pt^4+^ (15%) were found. After the photocatalytic measurement, the Pt 4f profile of the two samples remained qualitatively similar: In both cases a significant reduction to the metallic state occurred but a notable amount of Pt^2+^ was still detected. However, the higher stability of Pt^2+^ on the PA sample is again observable; the Pt^2+^ content of the recovered PAPtCalc (line “used” in Figure 7A) is clearly higher than that of the P25PtCalc (line “used” in Figure 7B).

The Pt/Ti atomic ratio in the fresh PAPtCalc sample was about 0.008, which did not change during the photocatalytic reaction. However, the Pt/Ti atomic ratio in the fresh P25PtCalc sample was significantly higher (0.012) with respect to the fresh PAPtCalc sample. 

In terms of % weight, the Pt content of the PA supported sample was around 1.5%, while in the P25 supported sample, it was approximately 2.5%. The Pt content higher than the nominal value is related to the surface location and probably good dispersion of Pt. The Pt/Ti atomic ratio in the recovered P25PtCalc sample decreased somewhat to 0.009, which was likely due to a slight decrease in Pt dispersion.

**Figure 8:**
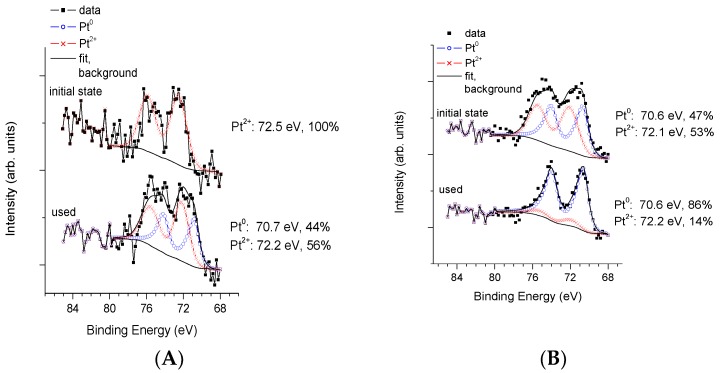
XPS study of fresh and recovered sample pairs in case of co-catalyst formation in high temperature nitrogen flow: (**A**) PA; and (**B**) P25.

Figure 8 summarizes the XPS results obtained on the chemical state of platinum in case of high temperature N_2_ treatment. It can be seen that the amount of the oxidized and metallic forms of Pt is completely different in fresh PAPtN_2_tr and in fresh P25PtN_2_tr (cf. patterns “initial state” in Figure 8A,B): Metallic Pt is absent from the former, but accounts for almost 50% of the Pt content of the latter. This observation means that autoreduction, i.e., reduction of Pt by NH_3_ species released from the precursor during annealing in inert gas is effective only for the P25-based sample. Regarding the effect of the photocatalytic reforming reaction on the co-catalyst, the Pt was almost completely in metallic state in the recovered P25PtN_2_tr sample (86%), while there was a mixture of metallic (44%) and Pt^2+^ (56%) forms in nearly equal ratio in the recovered PAPtN_2_tr. Beside this, the Pt/Ti ratio was slightly smaller in PAPtN_2_tr (0.008) than in P25PtN_2_tr (0.010), as already seen in case of the calcined samples. In terms of % weight, the PA supported sample contained 1.4% Pt, while in the P25 supported sample, this value was approximately 1.6% in the initial state. During the photocatalytic reforming of glycerol, the Pt/Ti ratio of the P25 TiO_2_ sample slightly decreased while no changes were seen in the PA supported sample.

In summarizing the XPS results, it could be said that in situ reduction of Pt occurs not only in photocatalytic methanol reforming [29], but also under the conditions of photocatalytic glycerol reforming. In addition, the general behavior of the PA and the P25 supported samples was similar, however, two clear differences between the supports were repeatedly observed: (i) Slightly higher amount of Pt was present in the P25 supported samples than in the PA supported ones; and (ii) Pt was more oxidized both in the fresh and the recovered PA supported samples, than in the P25 supported samples.

**Figure 9:**
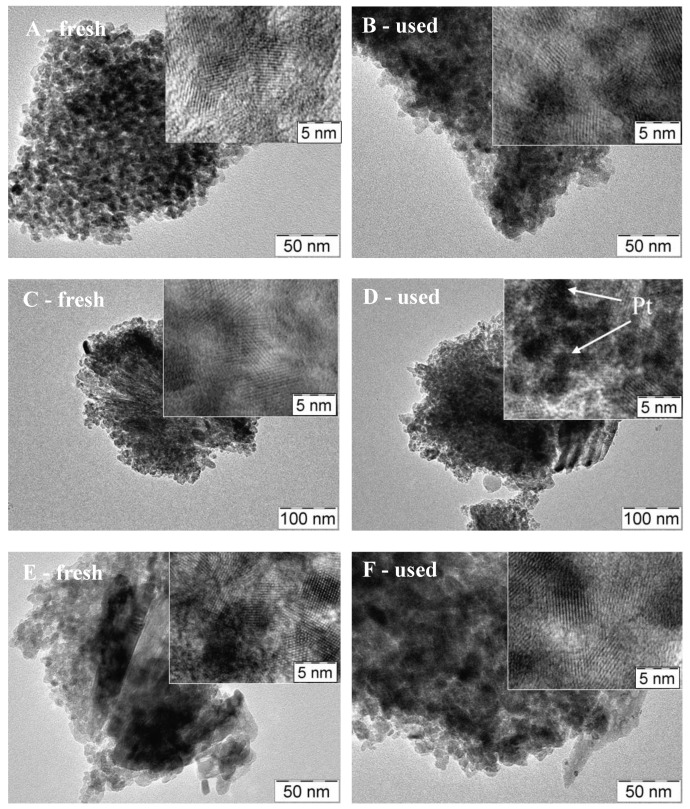
TEM images of fresh and recovered PAPt photocatalysts: (**A**,**B**) PAPtH_2_red; (**C**,**D**) PAPtCalc; and (**E**,**F**) PAPtN_2_tr. Pt containing nanoparticles are indicated by arrows.

**Figure 10:**
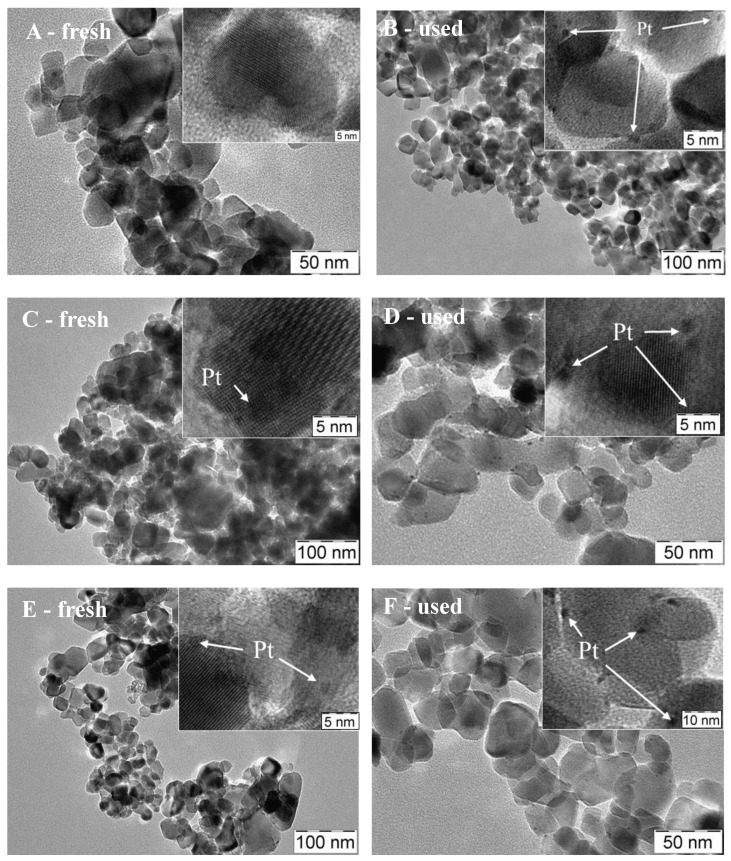
TEM images of fresh and recovered P25Pt photocatalysts: (**A**,**B**) P25PtH_2_red; (**C**,**D**) P25PtCalc; and (**E**,**F**) P25PtN_2_tr. Pt containing nanoparticles are indicated by arrows.

In Figure 9 and Figure 10, a comparison among TEM images of fresh and used photocatalysts is reported. It is quite clear that the low metal loading (1 wt %) favors the Pt dispersion and that the detection of particles is possible only at high magnification. By starting from the fresh PAPtH_2_ red sample, (Figure 9A), it can be seen that the catalyst is composed of well-aggregated TiO_2_ particles of 15–10 nm in size. 

Even at high magnification, it was not easy to observe Pt particles in a well-defined shape. After the photocatalytic test (see Figure 9B), the morphology of TiO_2_ remains very similar to that observed in the fresh sample, while the Pt particles become visible at high magnification even if they are very small (<0.5 nm). The PAPtCal catalyst is more compact than the reduced one and at high magnifications it is possible to observe the Pt particles even if they are present in the oxidized state. On the contrary, during the reaction, the Pt is reduced and the particles are clearly visible with variable dimensions in the 2–4 nm range. Therefore, calcination seems to favor the enlargement of the Pt particles that tend to aggregate. Regarding the treatment in N_2_, no significant difference was noticed with respect to the calcined catalyst, neither in terms of morphology, nor in the size of the Pt particles, which remain very small even after the reaction.

The samples prepared by impregnation of the commercial TiO_2_ (samples P25Pt) differ from the PAPt samples (Figure 10). 

In particular, the TiO_2_ particles are larger (20–100 nm) and less porous. On the fresh P25PtH_2_ red sample (Figure 10A), the Pt particles are too small and are not clearly visible even at high magnification. However, after the reaction, the metal particles were easily detected (1–2 nm in size) and resulted to be well distributed (Figure 10B). Most likely during the reaction, a slight sintering occurred, or the particles segregated on the surface so they appear more visible. Neither the morphology of the TiO_2_, nor the size of the particles of Pt that varies in the range 1–3 nm both on the fresh or the used samples seem to be significantly modified in case of the calcined samples (Figure 10C,D). The same behavior is observed on the sample treated in N_2_ (Figure 10E,F), and also, in this case, the Pt particles do not change in distribution and size (2–3 nm), both on the fresh and the used sample.

### 3.4. Effect of Water on the Autoreduction of Pt(NH_3_)_4_(NO_3_)_2_

**Figure 11:**
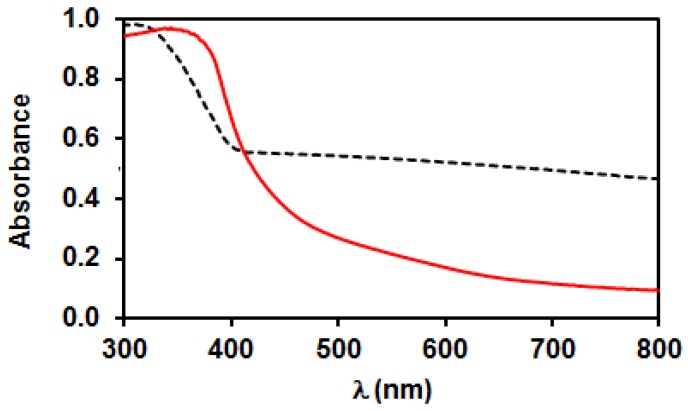
Diffuse reflectance UV-Vis spectra of the PtO_x_-TiO_2_ samples obtained by heat treatment in N_2_ flow (autoreduction). Solid line: PA; dashed line: P25.

As already discussed in the introduction overview and as confirmed by the XPS results, the heat treatment of Pt(NH_3_)_4_(NO_3_)_2_ could lead to autoreduction of platinum in certain cases. In spite of the same drying procedure, the photocatalysts obtained after decomposition of Pt(NH_3_)_4_(NO_3_)_2_ in N_2_ atmosphere on the two supports exhibited clearly different appearance. The color of the P25PtN_2_tr sample was grayish, while that of PAPtN_2_tr was brownish yellow. The diffuse reflectance UV-vis spectra of the N_2_ treated samples well demonstrate this difference (Figure 11), which can be explained by the presence of metallic Pt in the P25 supported case. The XPS measurements already indicated that the ratio of Pt^0^ is much higher in the fresh P25PtN_2_tr than in fresh PAPtN_2_tr (cf. patterns “initial state” in Figure 8A,B). 

**Figure 12:**
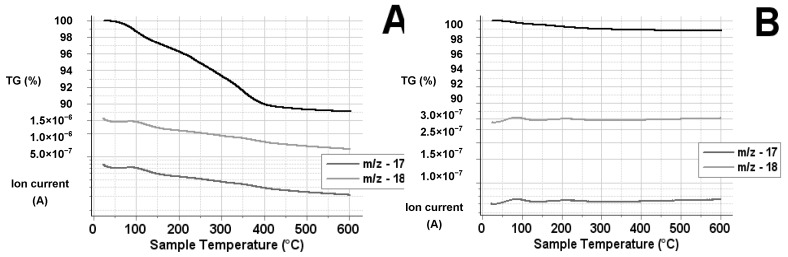
TG-MS trace of “blank“ impregnated TiO_2_ samples: (**A**) PA; and (**B**) P25.

Since, according to the literature data, during the thermal breakdown the water could significantly influence the formation of the platinum nanoparticles [60], such difference could be ascribed to different water retention of the two parent TiO_2_s. ^1^H MAS NMR results also had indicated significant difference in the distribution of surface -OH groups (and adsorbed water) on the two bare TiO_2_ materials (Section 3.3). Thus, to study the water adsorption capability of TiO_2_ samples, “blank” impregnated samples were prepared (using incipient wetness with pure water then followed by the same drying procedure which was used for Pt loading) and their thermal behavior was evaluated by TG-MS technique. According to the results shown in Figure 12 and in close correspondence to the behavior described in the literature [81], the water removal from both TiO_2_ materials occurred in two main steps: Molecularly adsorbed species were released up to 150 °C, while mass loss between 150 and 400 °C can be ascribed to the desorption of chemisorbed species (i.e., OH groups).

In case of PA a mass loss (Δm) of 3.1% was observed in the desorption range of molecular water; the chemisorbed species desorbed in a broad, continuous range between 150 and 400 °C (Δm 7.8%), which gave a total mass loss of 10.9%. On the contrary, the total mass loss was 1.2% from the “blank” impregnated P25 sample. Data on Figure 12 therefore confirm that the homemade PA TiO_2_ retained much more water than P25 after the same drying procedure. While homemade PA had smaller pore volume than P25 (see Table 1), the maximum pore diameter measured on PA was almost one order of magnitude less than that measured on P25 (3.7 nm vs. 31.5 nm), which could explain the higher water retention of PA type TiO_2_ during the introduction of the platinum precursor, in addition to its higher SSA value.

**Figure 13:**
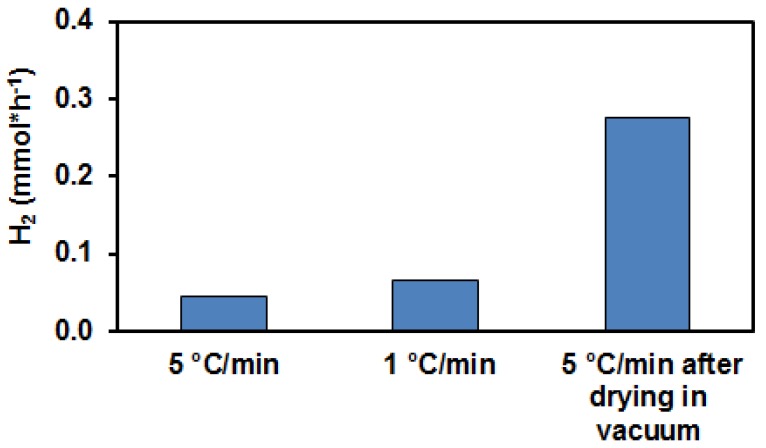
Dependence of the photocatalytic H_2_ production over PAPtN_2_tr samples on the water removal after the impregnation step. Reaction time: 270 min.

In order to force the water removal from PA based samples during the formation of the co-catalyst, the heating rate of the N_2_ treatment was decreased to one fifth (1 °C/min), as strongly reduced heating rate was found to be helpful in the case of zeolites [69]. In another approach, the impregnated sample was kept in a vacuum oven at 160 °C for 6 h before heating it up to 400 °C in N_2_ flow. The photocatalytic behavior of these samples was studied and the results are shown in Figure 13.

It can be seen that the effect of the reduced heating rate on H_2_ production was only marginal, whereas the vacuum pre-treatment significantly contributed to the increase of the hydrogen production. 

**Figure 14:**
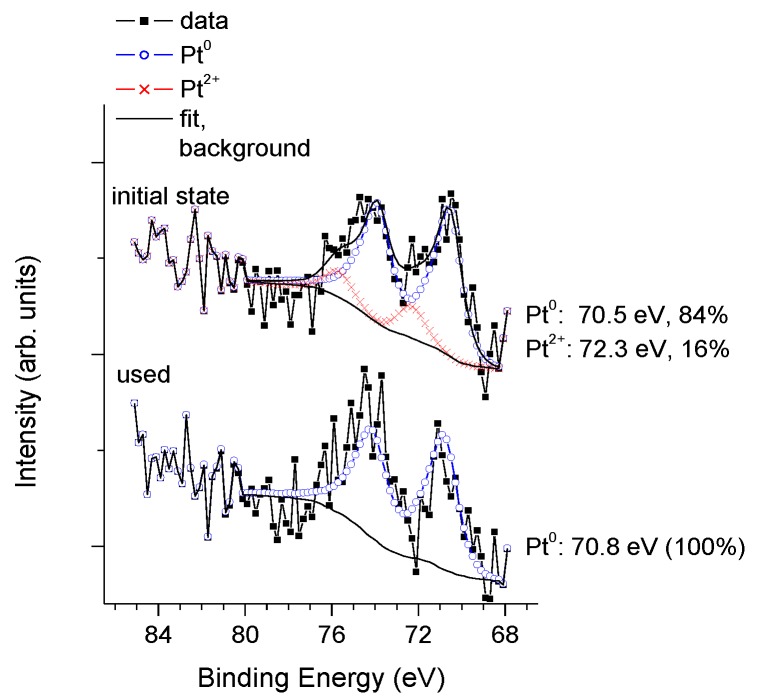
XPS study of the vacuum-dried PAPtN_2_tr sample.

The Pt 4f XPS profiles of the vacuum-dried PAPtN_2_tr sample in the fresh and recovered states are shown in Figure 14.

**Figure 15:**
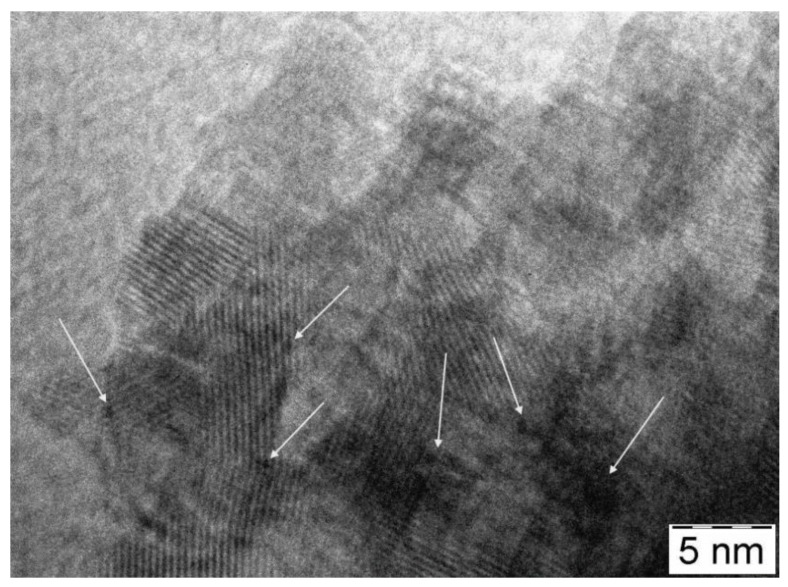
TEM image of recovered vacuum-dried PAPtN_2_tr sample. Pt containing nanoparticles are indicated by arrows.

The data reveal a very different situation from that observed for the non-vacuum-dried case (Figure 8A). Vacuum drying prior to the autoreductive treatment resulted in almost complete reduction of Pt, even in the fresh sample (cf. patterns “initial state” in Figure 8A and Figure 14). At the same time, no ionic Pt was found in the recovered sample (cf. patterns “used” in Figure 8A and Figure 14). This increase in the metallic nature of the co-catalyst should be the reason of the increase in the rate of hydrogen evolution. The low but stable Pt/Ti ratio (0.003) indicated a similar structure for Pt as it was in the case of PAPtH_2_ red, with the suggestion being supported by the TEM image of vacuum-dried PAPtN_2_tr sample (cf. Figure 9A and Figure 15).

## 4. Discussion

In this work, two different TiO_2_ supports were used to prepare photocatalysts for H_2_ production reaction by glycerol reforming. These semiconductors differed from each other in SSA values, pore volumes, maximum pore diameters, phase composition, and accordingly in surface and defect site structure. Thus, it is not surprising that photocatalysts prepared after Pt deposition showed widely differing properties both in their structure and activity. In this section, we attempt to identify some of the key features explaining these differences.

One of the most obvious differences between the PA and P25-supported samples was the apparently different Pt/Ti ratio measured by XPS, even if the real Pt content was almost the same as confirmed by the ICP-OES data. Considering that the TEM measurements confirmed very high dispersion of Pt in both cases, it could not explain the different Pt/Ti ratios. A more probable interpretation is related to the structural model behind the evaluation of the XPS data. Namely, in this work, a homogeneous depth distribution model for Pt and TiO_2_ was assumed. This approach naturally overestimates the Pt content if well-dispersed Pt particles decorate the surface of much larger TiO_2_ particles. Such a situation occurs in the case of the P25 supported photocatalysts. However, if the size of the Pt and the TiO_2_ particles are more comparable, the homogeneous model becomes more valid and the compositional results are closer to the real values, as observed for the PA supported photocatalysts. Therefore, the observed composition difference between the two supports could not reflect a real difference in the structure of Pt, rather it is related to a geometrical effect. A difference in encapsulation by TiO_2_ [82] could also be taken into account, but this process is generally relevant in the reduced cases and at temperatures much higher than those used in this study.

Regarding the polymorphs of titania, usually anatase is considered to be the “best” catalyst in the general photocatalysis literature [83]. At the same time, the flame-synthesized P25 TiO_2_ containing anatase, rutile, and some amorphous phase is regarded as “the standard photocatalyst” due to its relatively high activity in a multitude of reactions [84]. It is generally believed that the exceptional properties of the P25 titania can be connected to a beneficial interaction between its anatase and rutile content. However, there exist reports attributing higher activity to certain rutile-based TiO_2_ formulations than to their anatase-based counterparts in photocatalytic methanol reforming [23,85]. In the latter work, a comparison between methanol dehydrogenation on Pt-loaded P25 and rutile or anatase separated from P25 challenged the idea of beneficial inter-polymorph charge transfer as an explanation for the outstanding properties of the P25 support.

These ambiguities suggest that the bulk phase composition may not be the only decisive factor determining the photocatalytic properties of a given TiO_2_ formulation. It is plausible since heterogeneous photocatalysis is predominantly a surface chemical reaction for which a strong reductant/oxidant is available in the form of the photoexcited charge carriers, which were created and transferred to the reaction site by bulk processes. In other words, the surface state of the photocatalyst (order-disorder, defect structure, and bonding, chemical state and structure of the co-catalyst) should also be considered.

In fact, one may assume that the higher SSA of the PA should result in better overall photocatalytic performance. It is also believed that the smaller primary TiO_2_ particle size gives better photocatalytic activity [86]. Considering these properties, a performance at least comparable to that of the P25-supported catalysts could be expected in the case of the PA support.

Since the hydrogen generation over the bare supports was below the detection limit of our experimental setup, direct comparison of the catalytic properties of the bare titania materials was not feasible in this study. Nevertheless, comparison of the activity of the hydrogen treated PA and P25 supported sample pair still allows, to some extent, the assessment of the effect of the properties of the support on the catalytic performance. It is well established that in the photocatalytic reforming reactions over Pt/TiO_2_ catalysts, the metallic state of platinum is the most active because metallic Pt is a better catalyst for proton reduction and H_2_ formation then partially oxidized Pt [87]. As, according to our TEM and XPS experiments, the state of Pt was comparable for the PAPtH_2_red–P25PtH_2_red pair, the difference in the H_2_ production activity could be related mostly to the differences in the TiO_2_ semiconductor. 

In our experiments, consistently inferior hydrogen production data were obtained for the PA-supported catalysts, involving the hydrogen treated pair. This observation indicates that certain microscopic features counterbalanced the effects of the larger SSA or the smaller primary particle size in the case of the PA support.

One of these features is certainly the pore distribution. In the case of the PA support, a significant surface area arises from the inner walls of the quite small pores, which are water saturated in the as synthesized state, so neither Pt deposition during co-catalyst preparation nor penetration of reactants under the reaction conditions is easy, thus these areas may remain inactive during the photocatalytic reaction. 

Another important factor is the defect structure of the semiconductors. ESR indeed indicated clearly higher amounts of defects for the bare PA support compared to the P25 support. TEM images suggested higher levels of structural disorder for the PA support (smaller, more irregular, often partially sintered primary particles). At least a certain fraction of the defects observed by ESR should arise from these disordered regions at which formation of deep electron traps/recombination centers is expected, so the more defective structure of the PA support obviously interferes with its catalytic properties.

At the same time, surface defects act as nucleation sites for Pt particles. The higher amount of defects in case of the PA support may result in higher Pt dispersion. Indeed, TEM data obtained on the fresh P25PtCalc and the PAPtCalc sample pair activated by calcination suggested smaller Pt particle size on the PA support (≤1 nm in PAPtCalc and about 1–3 nm in P25PtCalc). A similar situation could be observed in case of the sample pair obtained by the high temperature nitrogen treatment; while after treatments in hydrogen, both on PA and on P25, particularly highly dispersed state of Pt was found.

XPS investigations carried out on the calcined sample pair revealed a completely oxidized state (Pt^2+–4+^ mixture) for Pt in the fresh catalysts, while a certain extent of in situ reduction to the metallic state was observed during the photocatalytic process. This in situ reduction is analogous to the reduction of ionic Pt during photodeposition [37], but there is an important difference in the initial state of Pt: In photodeposition, adsorbed Pt ions are reduced while in our case reduction of oxidized Pt nanoparticles occurs, thus the extent of the in situ reduction depends on the structure and stability of the nanoparticles. The fact that, in spite of the in situ reduction, the calcined one gave the smallest hydrogen yield among all P25 supported samples, while hydrogen evolution on the calcined PA supported photocatalyst was almost negligible confirms that oxidized Pt is not beneficial in glycerol photocatalytic reforming.

Comparison of the performance and properties of the nitrogen-treated sample pair (PAPtN_2_tr and P25PtN_2_tr) with those of their calcined and hydrogen-treated counterparts reveals another important aspect of the co-catalyst formation. Namely, our XPS experiments with the calcined and the nitrogen treated samples consistently indicated that Pt is more oxidized on the PA support than on P25, both in the as prepared and the recovered state. Even if some uncertainty in the oxidation state of the metal is unavoidably present as existence of Pt in rather dispersed form always involves the possibility of re-oxidation during the sample handling, the data suggest that reduction of Pt on the PA support is hindered in comparison to the P25 support. For example, when the co-catalysts were formed by N_2_ treatment, the state of the Pt in the two fresh catalysts was completely different (completely oxidized (Pt^2+^) for PAPtN_2_tr and partially metallic for P25PtN_2_tr) from each other. The state of Pt in the fresh PAPtN_2_tr therefore resembled that of the fresh PAPtCalc. In line with this, the difference between the activities of samples PAPtCalc and PAPtN_2_tr was negligible, which can also be related to the similarity in their Pt^0^ content after recovery (40% vs. 44%; essentially equal within the error limits of the XPS measurement). On the contrary, there was a big difference between the activity of samples P25PtCalc and P25PtN_2_tr in favor of the latter. This observation was consistent with the fact that the nitrogen annealing resulted in significant Pt^0^ content even in the fresh P25PtN_2_tr (47%), which increased up to 86% in the recovered sample as a result of the in situ reduction, while the P25PtCalc turned out to be more resistant towards the in situ reduction (only 55% Pt^0^ in the recovered sample).

In fact, the activity of the P25PtN_2_tr sample was close to that of the reduced P25PtH_2_red case, which indicates that autoreduction (reduction by NH_3_ species released from the Pt precursor during annealing in inert atmosphere [69]) may be as effective as reduction by H_2_ in achieving good performance. The question is why autoreduction of the platinum precursor was successful for the P25-based sample and why it did not work for the PA-based sample during the high temperature N_2_ treatment. Our experiments pointed out that the answer should be the high water retention capacity of the PA TiO_2_, as proven by the TG-MS measurement: We found that after an additional vacuum treatment at 160 °C before the high temperature N_2_ treatment, the photocatalytic activity on PAPtN_2_tr (0.28 mmol × h^−1^) was similar to that on the PAPtH_2_red sample (0.31 mmol × h^−1^). This observation confirmed the importance of the water removal for the success of the autoreduction after the impregnation with Pt(NH_3_)_4_(NO_3_)_2_ solution, which is not needed for the P25 support with much lower water retention.

According to our knowledge, the literature has reported the negative impact of the excess water only in the case of zeolite support when [Pt(NH_3_)_3_(H_2_O)]^2+^, [Pt(H_2_O)_x_]^2+^, and bare Pt^2^^+^ was formed step by step during the temperature programmed pretreatment with oxygen if the sample was not dried [62]. In related studies, it has been pointed out that interaction between the metal precursor, the supporting oxide and ambient gas is markedly different for neutral supports and acidic HY zeolites [60]. During the calcination of [Pt(NH_3_)_4_]^2+^ in zeolites, Pt^2+^ ions after losing their NH_3_ ligands, can either by oxidized with O_2_ to Pt^4+^ (in HY) or react with H_2_O resulting in formation of PtO microcrystallites (in NaY) [60]. The above literary results may suggest that the higher amount of acidic OH groups on PA TiO_2_ can lead to the increased Pt^4+^ content on the PA-based samples after co-catalyst formation by calcination.

Based on these observations, the water retention capacity of the bare TiO_2_ should be considered as a key factor considering that the decomposition of Pt(NH_3_)_4_(NO_3_)_2_ platinum precursor is strongly influenced by the presence of water. We believe that the more stable Pt-oxide species found on the PA support form, at least partly, because of the initial interaction of the Pt precursor with the abundantly available water during the co-catalyst formation treatment.

To summarize the discussion, our studies confirmed that the behavior of a photocatalyst cannot be predicted only by knowing the phase composition/SSA/grain size of the semiconductor. Instead, the interplay between the photoelectric properties of the semiconductor, its surface properties, and its interactions with the co-catalyst, all influenced by the semiconductor preparation and co-catalyst deposition and activation methods should be considered.

## 5. Conclusions

In this study, 1 wt % Pt/TiO_2_ photocatalysts were prepared for photocatalytic reforming of glycerol on two very different supports, on P25, and on home-made high surface area TiO_2_ (PA). Three different methods (reduction in H_2_, calcination in air and heat treatment in N_2_) were used for activation of the Pt co-catalyst. The activity of photocatalysts based on P25 TiO_2_ was much higher than that of the PA based ones in all cases. The difference strongly depended on the nature of the co-catalyst nanoparticles, which was influenced by both the method of the co-catalyst formation and by the semiconductor.

Regarding the three co-catalyst formation methods, the reduction of the Pt(NH_3_)_4_(NO_3_)_2_ as platinum precursor in the H_2_ atmosphere at elevated temperature was more favorable than the other co-catalyst formation methods. Co-catalyst formation by calcination gave the worst activities. The extent of in situ reduction of the platinum co-catalyst, formed by calcination, was different in case of the different TiO_2_ supports; a higher amount of metallic Pt was observed in the more active P25-supported sample. Oxidized Pt does not seem to be a good co-catalyst in photocatalytic glycerol reforming.

In order to get well dispersed Pt^0^ nanoparticles from Pt(NH_3_)_4_(NO_3_)_2_ by means of autoreduction in nitrogen, the impregnated titania must be carefully dried because water plays a decisive role in the decomposition of this platinum compound, not only in the case of zeolites, but in the case of TiO_2_ supports, too.

## Figures and Tables

**Table 1:** Main structural properties of the studied TiO_2_.

Type of TiO_2_	SSA ^1^, m^2^ g^−1^	Maximum Pore Diameter, nm	Pore Volume, cm^3^ g^−1^	Crystalline Phases ^2^, %			Average Particle Size, nm		
				A ^3^	B ^4^	R ^5^	A ^3^	B ^4^	R ^5^
PA	133.8	3.71	0.1064	23	23	54	6 ^2^	7 ^2^	19 ^2^
P25	52.5	31.55	0.3674	82	-	18	24 ^2^	-	45 ^2^

^1^ Specific surface area (SSA) calculated from BET measurements; ^2^ Calculated from the XRD measurements; ^3^ A: anatase; ^4^ B: brookite; ^5^ R: rutile.
